# Investigation of mechanisms underlying chaotic genetic patchiness in the intertidal marbled crab *Pachygrapsus marmoratus* (Brachyura: Grapsidae) across the Ligurian Sea

**DOI:** 10.1186/s12862-020-01672-x

**Published:** 2020-08-24

**Authors:** A. Iannucci, S. Cannicci, I. Caliani, M. Baratti, C. Pretti, S. Fratini

**Affiliations:** 1 Department of Biology, University of Florence, via Madonna del Piano 6, 50019 Sesto Fiorentino, Italy; 2 The Swire Institute of Marine Science and the Division of Ecology and Biodiversity, The University of Hong Kong, Pokfulam Road, Hong Kong, Hong Kong SAR; 3 Department of Environment, Earth and Physical Sciences, University of Siena, via Mattioli 4, 53100 Siena, Italy; 4 National Research Council - IBBR, via Madonna del Piano 6, 50019 Sesto Fiorentino, Italy; 5 Interuniversity Consortium of Marine Biology of Leghorn “G. Bacci”, viale N. Sauro 4, 57128 Livorno, Italy; 6 Department of Veterinary Sciences, University of Pisa, via Livornese lato monte, 56122 San Piero a Grado (PI), Italy

**Keywords:** Microsatellites, Relatedness, Kin-aggregation, DAPC, Self-recruitment, Micro-geographic scale

## Abstract

**Background:**

Studies on marine community dynamics and population structures are limited by the lack of exhaustive knowledge on the larval dispersal component of connectivity. Genetic data represents a powerful tool in understanding such processes in the marine realm. When dealing with dispersion and connectivity in marine ecosystems, many evidences show patterns of genetic structure that cannot be explained by any clear geographic trend and may show temporal instability. This scenario is usually referred to as chaotic genetic patchiness, whose driving mechanisms are recognized to be selection, temporal shifts in local population dynamics, sweepstakes reproductive success and collective dispersal.

In this study we focused on the marbled crab *Pachygrapsus marmoratus* that inhabits the rocky shores of the Mediterranean Sea, Black Sea and East Atlantic Ocean, and disperses through planktonic larvae for about 1 month. *P. marmoratus* exhibits unexpectedly low connectivity levels at local scale, although well-defined phylogeographic patterns across the species’ distribution range were described. This has been explained as an effect of subtle geographic barriers or due to sweepstake reproductive success. In order to verify a chaotic genetic patchiness scenario, and to explore mechanisms underlying it, we planned our investigation within the Ligurian Sea, an isolated basin of the western Mediterranean Sea, and we genotyped 321 individuals at 11 microsatellite loci.

**Results:**

We recorded genetic heterogeneity among our Ligurian Sea samples with the occurrence of genetic clusters not matching the original populations and a slight inter-population divergence, with the geographically most distant populations being the genetically most similar ones. Moreover, individuals from each site were assigned to all the genetic clusters. We also recorded evidences of self-recruitment and a higher than expected within-site kinship.

**Conclusions:**

Overall, our results suggest that the chaotic genetic patchiness we found in *P. marmoratus* Ligurian Sea populations is the result of a combination of differences in reproductive success, *en masse* larval dispersion and local larval retention. This study defines *P. marmoratus* as an example of marine spawner whose genetic pool is not homogenous at population level, but rather split in a chaotic mosaic of slightly differentiated genetic patches derived from complex and dynamic ecological processes.

## Background

Many theoretical and empirical studies have shown that, in marine populations, connectivity plays a fundamental role in population and metapopulation dynamics, community dynamics and structure, genetic diversity, and the resilience of populations to human impact [1–5]. A comprehensive appraisal of the population dynamics of marine and intertidal organisms, however, can prove difficult even when genetic data are available. This difficulty often derives from a lack of exhaustive knowledge on the larval dispersal component of connectivity [6].

The observed spatial genetic structure of marine species seldom fits models developed for terrestrial populations, such as the island or the stepping-stone models [7–9]. In the sea, dispersal usually occurs throughout pelagic larval phases, which persist in offshore waters from few days to several weeks [6]. Surface currents and winds contribute to the dispersal of pelagic larvae and, ultimately, affect the connectivity of most populations. At fine geographical scales, linear distances may be a poor proxy of gene flow [10], since surface currents are highly turbulent and nonlinear [11, 12]. Early gene flow estimates for marine species only relied on simplified models based on one-way oceanic currents acting on passive particles [2]. The recently developed combined biological-physical models better describe gene flow estimates, since they also include the possible influence of biological factors such as rates of larval mortality and active vertical positioning behavior of larvae on dispersal trajectories [2].

Emerging evidences are showing that marine populations may be genetically structured over smaller spatial scales than was previously thought [13]. These new patterns are challenging our assumptions and, ultimately, our predictions about connectivity in marine species. Moreover, geographic distribution of population genetic divergence cannot be fully explained by a clear geographic trend and may show temporal variability. This scenario has been reported in many studies [12, 14–26] and it is usually referred to as Chaotic Genetic Patchiness (CGP, sensu [12]). CGP was defined as the spatial and temporal patterns of population genetic structure observed in marine fish and invertebrates at short scale considering the presumed dispersal range of planktonic larvae.

Understanding the mechanisms which drive CGP is still a challenge in marine ecology. Researchers suggest four main alternative mechanisms to explain CGP [13, 27–29]: selection, temporal shifts in local population dynamics, sweepstakes reproductive success and collective dispersal.

First, natural selection may act on larvae in a pre- or a post-settlement phase, by means of severely fluctuating environmental conditions that could drive selection for locally beneficial alleles through differential survival of recruits [12, 30]. CGP driven by post-larval settlement selection is particularly important on rocky shores, where emersion time and fixation on the substrate strongly vary with intertidal position [31–33]. Pre-settlement selection plays a crucial role also in benthic marine species, whose settlement and recruitment success are influenced by the ability to reach suitable substrata [34].

Second, CGP may be driven by temporal variations in the cohorts of recruits with respect to the source populations, with juveniles that recruit in a given population possibly coming from different sources at different times. Seasonal or inter-annual changes in currents as well as asynchrony in spawning events across populations may operate a stochastic selection on planktonic larvae and favour recruits from different sources [24, 28, 35]. Thus, mixing, genetically differentiated larval pools may lead to variation in the genetic composition of recruits, resulting in unpatterned genetic heterogeneity among populations. This condition is usually met in semelparous species, but it can be found also in species with overlapping generations, if parents and offspring are spatially well separated [36, 37].

Third, the high variance in reproductive success typical of marine organisms, known as sweepstakes reproduction, can also greatly contribute to reduce the effective size of local breeding groups and, consequently, it might explain the reduced genetic variation within cohorts of larvae and new recruits that represent the reproductive output of a minority of adult individuals (sweepstake reproductive success hypothesis: [19]; reviewed in [38]). This is supported by several genetic studies that elucidated parentage and relatedness in wildlife populations and compared genetic diversity of adults and offspring [24, 25, 29, 39, 40].

At last, collective dispersal indicates any process leading to gene flow by groups of individuals [41]. It can be described as any dispersal process where pairs of immigrants in the same population have a higher than random chance of having originated from the same natal population. This type of dispersal may arise from individual dispersal strategies, such as collective larval dispersal by ocean currents, if pools of larvae released from a local breeding group do not diffuse randomly but remain aggregated to some extent during dispersal and settlement [27, 42].

The marbled crab *Pachygrapsus marmoratus* is an intertidal brachyuran crab that inhabits the rocky shores of the Mediterranean Sea, Black Sea and East Atlantic Ocean. The population genetic structure of this species has been studied at both regional (using hypervariable nuclear markers: [43–49]) and macro-geographic scales (using the cytochrome c oxidase I gene: [43, 44, 46, 48, 50]). Across its entire distribution range, Fratini et al. [50] identified three genetically differentiated groups, corresponding to the Portuguese Atlantic Ocean, the Mediterranean Sea plus Canary Islands, and the Black Sea. When population structures were investigated at small geographical scales [44, 45, 47, 49], unexpectedly low levels of connectivity were recorded, with no clear relation to any known geographic boundary. The above studies [44, 45, 47, 49] mainly linked the lack of relationship between geographical features and population structures to sweepstakes reproductive success, one of the mechanisms underlying the CGP, although they could not rule out environmental factors acting as subtle barriers in influencing larval dispersion.

The present study was specifically planned to assess, for *P. marmoratus*, the possible occurrence of a structure not related to geographic features at local scale, in order to depict a more defined CGP scenario and to ascertain the drivers underlying it. Following Pascual et al. [51], *P. marmoratus* life history traits make it a good candidate for local unpatterned population genetic structure. Adults are low-mobility benthic crabs with a restricted home range [52]. The species is also characterized by a pelagic larval period of medium duration (i.e. about 4 weeks) [53].

**Fig. 1:**
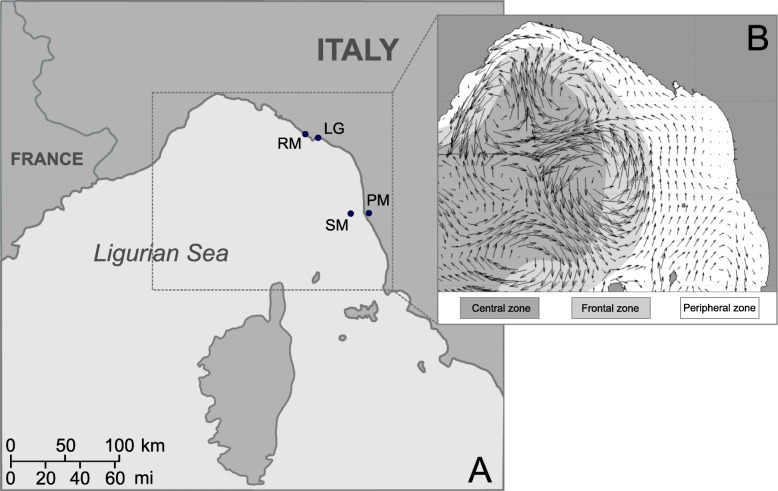
**a**, map of the study sites (modified from https://commons.wikimedia.org/wiki/File:Map_of_Italy-it.svg with Inkscape v0.92). *Pachygrapsus marmoratus* individuals were sampled for genetic analyses at four sites of the Ligurian Sea: Porto Mediceo (PM), Secche della Meloria (SM), Riomaggiore (RM), Le Grazie (LG). **b**, LIME-ROMS monthly mean of the surface horizontal velocity, vertically averaged in the first 20 m of depth (modified from Sciascia et al. [55] with Inkscape v0.92). The three main areas of the Ligurian basin are also shown (central zone, frontal zone, peripheral zone)

We selected four close sampling sites in an isolated basin of the Mediterranean Sea, the Ligurian Sea, to exclude from the analysis any subtle geographic barriers, such as variation in water temperature and salinity, that might have driven population structure at local scale. We sampled a total of 321 *P. marmoratus* individuals from the four sites and we genotyped them at eleven microsatellite loci. In comparison to previous studies [43–49], we collected a higher number of individuals per sites (about 80) in order to avoid both the underrepresentation of genetic diversity in local populations and a high influence of chance on the results. The selected area is the northernmost sector of the western Mediterranean basin and its peculiar hydrodynamic and meteo-oceanographic features separate it from the Tyrrhenian sea [54]. The horizontal circulation in the Ligurian Sea, the Ligurian Current, has a cyclonic aspect and is dominated by a geostrophic flow parallel to the coast, which enters from Northwest of the Corsica Island. It forms a permanent and robust flow area (frontal zone) that separates the coastal area (peripheral zone) and the inner area of the basin (central zone) (Fig. 1). The peripheral zone is less stable in structure than the central zone, as it is traversed by transient flows influenced by the effects of surface fluxes. Currents in the coastal area, however, are mainly flowing northwards [56].

## Results

### Genetic diversity

The analysis performed with MICROCHEKER indicated an excess of homozygote at the four sites (Porto Mediceo of Livorno, PM; Secche della Meloria, SM; Le Grazie, LG and Riomaggiore, RM). Since we found a significant positive relationship between locus specific sample deviations from the expectations of Hardy-Weinberg equilibrium (HWE) and the number of individuals that failed to amplify at each locus (F_(1, 38)_ = 25.41, *p* < 0.001), our data supported the hypothesis of null alleles for explaining these patterns. Thus, we corrected our dataset for null alleles following the INA method described in [57].

When assessing linkage disequilibrium among loci over the entire population, we always recorded a correlation coefficient *r*^2^ close to zero for all loci pairs (Table S1). The highest *r*^2^ value was recorded for the locus pair Pm-183 and Pm-6 (*r*^2^ = 0.17: Table S1). Since a threshold *r*^2^ value above 0.8 has been used to indicate a non-random association between the alleles present at two genetic loci [58], all loci were retained for subsequent analysis regardless of the significance level of the test (Table S1).

All loci were polymorphic (except locus Pm-86 that was excluded from the analyses), with number of alleles ranging from 3 (locus Pm-79) to 48 (locus Pm-16). Relatively high levels of genetic variability were recorded at all sampling sites (Table 1). Overall, allelic diversity and average allelic richness were similar for all sampling sites, ranging from 16.20 ± 4.08 to 18.10 ± 3.84 and from 15.84 ± 3.99 to 17.66 ± 3.75, respectively (Table 1). The total number of private alleles per sampling site ranged from 10 to 15 with the highest number assessed in SM (Table 1). The highest values of expected and observed heterozygosity were recorded for SM (Table 1). No sampling site deviated from HWE after Bonferroni correction, except PM at locus Pm-108, RM at locus Pm-101 and LG at locus Pm-108 (Table 1).

**Table 1:** Genetic diversity measures in *Pachygrapsus marmoratus* from four sampling sites in the Ligurian Sea. For each site, the GPS coordinates and *N,* number of analysed individuals, are reported. *N*_A_, number of alleles; *A*_R_, allelic richness; *A*_P_, number of private alleles; *H*_O_, observed heterozygosity; *He*, expected unbiased heterozygosity. Means ± SE values. *, *P* < 0.005 (after Bonferroni correction)

	Locus	79	109	101	108	183	99	16	187	84	6	Overall
**Porto Mediceo (PM)**	*N*_A_	2	40	17	5	14	20	35	16	2	11	16.2 ± 4.08
**43.549 N, 10.294 E**	*A*_R_	2.00	38.63	16.94	4.83	13.18	19.88	34.83	15.44	2.00	10.71	15.84 ± 3.99
	*A*_P_	0.00	2.00	0.00	0.00	1.00	2.00	7.00	1.00	0.00	0.00	13
***N*** **= 80**	*H*_O_	0.49	0.95	0.92	0.63	0.71	0.96	0.99	0.58	0.37	0.62	0.72
	*He*	0.50	0.96	0.87	0.64*	0.69	0.91	0.87	0.59	0.32	0.60	0.70
**Secche della Meloria (SM)**	*N*_A_	3	35	26	10	16	23	27	22	3	15	18.0 ± 3.37
**43.546 N, 10.218 E**	*A*_R_	2.87	34.90	25.10	9.61	15.27	22.72	26.78	21.10	2.86	14.52	17.57 ± 3.31
	*A*_P_	0.00	2.00	0.00	1.00	1.00	2.00	4.00	3.00	1.00	1.00	15
***N*** **= 81**	*H*_O_	0.53	0.99	0.89	0.68	0.79	0.96	0.97	0.77	0.41	0.73	0.77
	*He*	0.56	0.95	0.90	0.71	0.75	0.90	0.84	0.75	0.40	0.69	0.75
**Riomaggiore (RM)**	*N*_A_	3	39	27	9	13	26	30	17	2	15	18.1 ± 3.84
**44.096 N, 9.739 E**	*A*_R_	2.86	37.46	26.77	8.59	12.52	25.48	29.94	16.53	2.00	14.49	17.66 ± 3.75
	*A*_P_	0.00	1.00	3.00	1.00	0.00	1.00	1.00	1.00	0.00	2.00	10
***N*** **= 80**	*H*_O_	0.53	0.99	0.93	0.61	0.73	0.97	0.99	0.71	0.28	0.74	0.75
	*He*	0.50	0.96	0.92*	0.66	0.75	0.93	0.83	0.69	0.28	0.75	0.73
**Le Grazie (LG)**	*N*_A_	3	37	24	10	13	26	29	17	2	14	17.5 ± 3.60
**44.067 N, 9.841 E**	*A*_R_	2.87	36.01	23.76	9.49	12.60	25.31	28.89	17.00	2.00	13.32	17.12 ± 3.54
	*A*_P_	0.00	0.00	0.00	1.00	0.00	2.00	3.00	1.00	0.00	3.00	10
***N*** **= 80**	*H*_O_	0.52	0.96	0.96	0.62	0.80	0.96	0.97	0.67	0.41	0.74	0.76
	*He*	0.55	0.96	0.89	0.68*	0.80	0.91	0.83	0.68	0.41	0.73	0.74

### Population assignment and pairwise relatedness

Average pairwise relatedness based on the Lynch and Ritland estimator [59] for the four sampling sites were: PM, *r* = 0.003; SM, *r* = 0.000; RM, *r* = − 0.002; and LG, *r* = 0.000. All values except that of RM were significantly higher than the average relatedness calculated among all individuals across locations (*r* = − 0.002 ± 0.000, *P* < 0.05). Population assignment results indicated that individuals from all the four sites assigned to their own population with a probability always higher than 50% (PM, 76.25%; SM, 64.20%; RM, 53.75%; LG, 71.25%).

### Population structure

There was a weak genetic differentiation among sampling sites. While the Fisher exact test did not reject the hypothesis of genetic homogeneity of allele frequency distributions (probability of non-differentiation among populations *P* = 1.0), a weak but significant overall genetic differentiation among populations was recorded by *F*-statistics (θst = 0.008, *P* = 0.005). The variation among populations corresponded to 1%, while within population variation exceeded 99%. The pairwise comparisons showed a significant differentiation only between LG and PM and LG and SM (Table 2).

**Table 2:** Pairwise analysis of molecular variance (AMOVA) calculated among the four populations of *Pachygrapsus marmoratus* along the studied area. θst values (below the diagonal) and correspondent probability values (above the diagonal) are shown. Significance at *P* < 0.008 (after Bonferroni correction)

	PM	SM	RM	LG
**PM**	–	0.02	0.06	< 0.001
**SM**	0.007	–	0.04	0.006
**RM**	0.005	0.006	–	0.02
**LG**	0.016	0.009	0.007	–

**Fig. 2:**
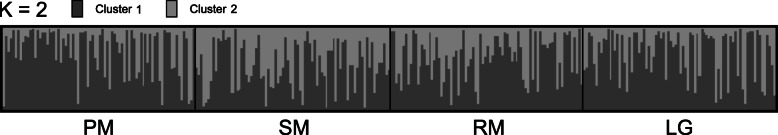
Clustering analyses for 321 *Pachygrapsus marmoratus* individuals performed using a Bayesian clustering approach (STRUCTURE). Each individual is represented by a vertical line partitioned into *K* segments, with lengths corresponding to the proportion of its genome originating from each of the *K* inferred clusters. Black vertical bars define distinct sampling sites. Site acronyms as in Fig. 1

The outcome of the Bayesian clustering approach implemented in STRUCTURE revealed that the most probable number of clusters for interpreting the observed genotypes was *K* = 2 based on the highest modal value of Δ*K* = 38.77 estimated using the Evanno et al. [60] method (Fig. S1a, Supporting Information). Four main partitions were used as a priori population information for calculating the posterior probability of individual assignment (Fig. 2). Individuals from all sites were generally assigned to both clusters 1 and 2. In particular, mean assignment probabilities to cluster 1 and 2 were 29 and 71%, 41 and 59%, 35 and 65%, 28 and 72% for PM, SM, RM and LG, respectively. Based on these values, populations PM and LG appear to be the most genetically similar ones, despite they are the most geographically distant ones.

**Fig. 3:**
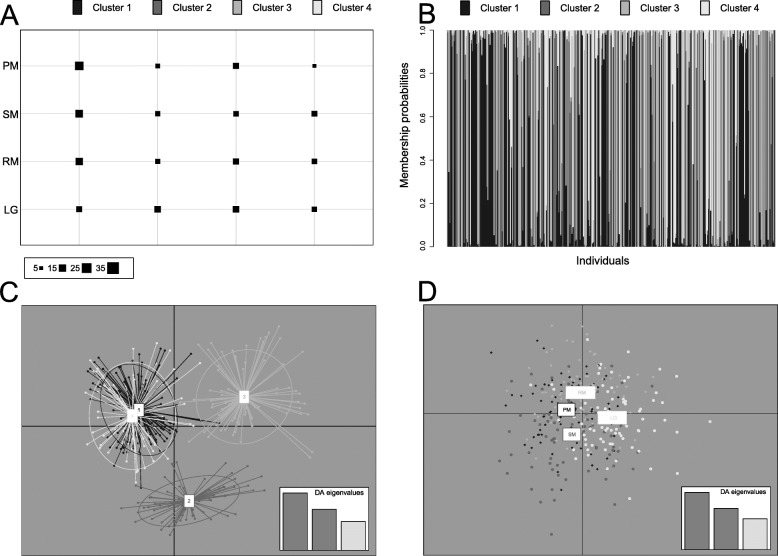
Summary of the results of discriminant analysis of principal components (DAPC) for 321 *Pachygrapsus marmoratus* individuals. **a**, number of individuals from each sampling site (vertical axis) assigned to each of the four inferred genetic clusters (horizontal axis). The size of black squares is proportional to the number of individuals assigned to each cluster (lower legend). **b**, DAPC compoplot showing the assignment of individuals to genetic clusters. Each individual is represented by a vertical bar, and colours indicate the probability of an individual’s membership in one of four genetic clusters. **c**, ordination plot for the first two discriminant axes. Dots represent individuals connected to the center of an inertia ellipsis, which indicates assignment to one of the four genetic clusters inferred by DAPC. **d**, DAPC with original sampling sites as clusters. Geographical origin of each population is depicted in the plot by colours and individuals are represented by dots. Location acronyms as in Fig. 1

The Discriminant Analysis of Principal Component (DAPC) suggested the presence of four distinct genetic clusters, as indicated by a rapid decrease of the Bayesian information criterion (BIC) values from K = 1 to K = 4 and a further minimal decrease for K > 4 (Fig. S1b, Supporting Information). Individuals from the four populations were almost equally distributed among all the four clusters suggesting a poor or nil genetic differentiation among original populations (Fig. 3a). The DAPC bar plot showed that the cluster subdivision did not match the original populations, as the individuals from each population were assigned to all the four clusters (Fig. 3b). The high overlapping of the genetic clusters on the ordination plot, especially between cluster 1 and 4, indicated low degree of differentiation between them (Fig. 3c). When the DAPC was run with geographic sites used as clusters, it showed low geographical-related differentiation among the four sites (Fig. 3d).

### Contemporary migration patterns

In order to detect source-sink dynamics among our Ligurian Sea populations, we estimated recent migration rates using a Bayesian approach as implemented in BayesAss. The outcomes of the analysis showed that the recent migration rates between pairs of sites were always very low, except when LG represented the outgoing site (Table 3). The proportion of self-recruitment within each locality was about 0.67 for PM, SM and RM and increased to 0.981 for LG (Table 3). These results are not in line with the main sea circulation pattern of the study area, which consists in a northward coastal current.

**Table 3:** Mean (± SE) and 95% confidence intervals (in parentheses) of Bayesian posterior distribution of recent migration rates between pairs of localities of *Pachygrapsus marmoratus*. Columns represent the outgoing migration rates and rows represent incoming migration rates. Bold values along the diagonal axis represent the proportion of non-migrants in each site

Into/from	PM	SM	RM	LG
PM	**0.671 ± 0.004 (0.663–0.679)**	0.004 ± 0.004 (0.000–0.011)	0.004 ± 0.004 (0.000–0.011)	0.321 ± 0.007 (0.308–0.334)
SM	0.004 ± 0.004 (0.000–0.012)	**0.672 ± 0.005 (0.663–0.681)**	0.004 ± 0.004 (0.000–0.012)	0.320 ± 0.008 (0.305–0.335)
RM	0.004 ± 0.004 (0.000–0.011)	0.004 ± 0.004 (0.000–0.012	**0.671 ± 0.004 (0.663–0.679)**	0.321 ± 0.007 (0.308–0.334)
LG	0.005 ± 0.005 (0.000–0.015)	0.008 ± 0.007 (0.000–0.021)	0.006 ± 0.006 (0.000–0.017)	**0.981 ± 0.01 (0.962–1.000)**

## Discussion

It is well-known that several marine organisms with high dispersal ability can show much greater spatial genetic heterogeneity over short distances than expected [61–64]. This study confirms that the intertidal crab *P. marmoratus* is an example of marine spawner that exhibits local genetic heterogeneity not related to geography and patterns of chaotic genetic patchiness.

A weak evidence of population structure is detected by Fst statistics, but not confirmed by the exact test of population differentiation. The outcomes of the Bayesian structure analyses and the DAPC show the presence of genetically differentiated clusters (two and four, respectively) not related to geography. The clusters identified by both approaches do not match the original populations, since the individuals from each site are assigned to all the genetic clusters. Overall, these results suggest that dynamic ecological processes act on *P. marmoratus* populations and cause a non-homogenous genetic pool at population level, which, ultimately, results in a mosaic of chaotic and slightly differentiated genetic patches. Another indication of chaotic genetic patchiness across the surveyed micro-geographic area comes from the slight differentiation among populations recorded by the Bayesian structure analysis, which found the geographically most distant populations to be the most similar ones from a genetic point of view.

In recording genetic heterogeneity at local scale, we first assessed an inter-population genetic divergence not in line with the geographical distance among our populations. We then recorded the occurrence of multiple genetic patches within each population, since our analyses grouped our specimens according to genetic clusters that did not match the four native populations. This may indicate that the larval pool is not homogeneously mixing off shore, due to local ecological factors (variations in sea water temperature and salinity as well as food availability [3]), or biological characteristics of the species (timing of reproduction and spawning as well as reproductive output [65–67]) that may trigger population dispersion and connectivity processes, as already advocated for other brachyuran species [68].

Across its wide distribution range, *P. marmoratus* is structured in few homogenous clusters in correspondence to the main phylogeographic barriers separating the Mediterranean Sea from the Atlantic Ocean and the Black Sea [50]. Conversely, local genetic heterogeneity was already reported in previous population genetic studies at regional-geographic scales. Silva et al. [49], for *P. marmoratus* sampled along the Portuguese coast, detected a genetic heterogeneity not related to geographic gradients, which they explained by means of coastal hydrological events influencing larval fluxes. Fratini et al. [45] recorded a partitioning of genetic variation among specimens collected along the seven islands of the Tuscan Archipelago and the mainland Tuscan coast (Mediterranean Sea), with all sites separated from each another, except the two southernmost islands, lying only a few kilometres apart from each other. Local larval retention and sweepstakes reproductive effect were ascribed as the main factors driving this pattern. Deli et al. [44] found population genetic differentiation across the Siculo-Tunisian Strait and a mixture of three genetic clusters within the eight African Mediterranean populations examined. Even this latter differentiation was neither associated with any geographic boundaries nor with geographic distances among the populations. Our results are thus in line with those of Silva et al. [49], Fratini et al. [45] and Deli et al. [44] in describing patterns of local genetic heterogeneity not related to geography. Our results also strengthen the presence of genetic clusters not corresponding to native populations in *P. marmoratus*, as already showed by Deli et al. [44], since our study is based on a higher number of microsatellites (10 compared to 4 loci) and individuals analysed per population (80 compared to 10–20). As discussed in the above-mentioned studies [44, 45, 49], both local larval retention and random reproductive success of small subsets of adults over time and space, which are two of the drivers advocated for a CGP scenario [13, 27–29], can play a crucial role in shaping such a genetic pattern in the *P. marmoratus* populations of the Ligurian Sea.

The novel evidence of the present study, mainly due to our intensive sampling approach, is therefore the high occurrence of self-recruitment and within-site kinship at the target sites. This may be driven by active larval behavioural mechanisms favouring local larval retention to natal sites [69, 70]. Self-recruitment might also be driven by the unstable circulation pattern within the Ligurian Sea peripheral zone, where local eddies, resulting from instabilities in the surface layer of the coastal current, may trap larvae for several days along the coast, particularly in inlets and bays, thus favouring retention to natal sites [71]. Another explanation may be the formation of aggregates of related larvae in the water column followed by *en masse* larval dispersion [72, 73]. Larvae of some marine spawners, linked by half-sib or full-sib relationships, were found to be transported in the same water mass and settle in close proximity to each other, thereby influencing the genetic structuring of the adult populations [24, 25, 74]. Broadly speaking, the formation of kin-aggregations, either due to self-recruitment or the formation of aggregates of related larvae, is an evolutionary process that deserves particular attention. At small spatial scales, in particular, the kin-aggregation can be associated with negative evolutionary costs, such as a reduction of gene flow and a potential increase of inbreeding rates [75, 76]. The pattern of CGP recorded in *P. marmoratus* populations from the Ligurian Sea could also be an indicator for a possible effect of natural selection acting on larvae. Indeed, the effect of post-settlement selection can be particularly relevant on *P. marmoratus* megalopae, which settle and grow into juveniles within the harsh and unstable intertidal zone characterised by strong daily and seasonal variations in salinity and temperature [77].

Future directions in marine ecology should address the mechanisms that affect regional patterns of recruitment and the events that drive post-settlement selection at a local scale, and, ultimately, investigate how kin-aggregation benefits may outweigh the costs of inbreeding on a small geographical scale.

## Conclusions

The observed presence of genetically differentiated clusters not corresponding to the native populations, coupled with a slight inter-population divergence not related to geography and a high percentage of individuals assigned to their own populations, strongly support a multiple set of drivers for the CGP observed in *P. marmoratus* from the Ligurian Sea. In particular, the present study suggests that mechanisms such as differences in reproductive success (sweepstakes reproductive effect), *en masse* larval dispersion, local larval retention and post-settlement larval selection may act individually in driving CGP in marine populations. Yet, we cannot exclude a complex and synergistic effect, especially when they contemporarily occur in a certain species and in a determined geographic context.

In this intertidal crab, genetic drift and non-random mating can also be advocated as key determinants for local genetic heterogeneity, since *P. marmoratus* is characterised by large breeding populations, sexual dimorphism towards larger males, high females’ fertility (over tens of thousands eggs per female per spawning event: personal authors’ data), and high dispersal potential. Moreover, natural populations of *P. marmoratus* are normally formed by individuals belonging to different generations and recruited during different events of settlements. We cannot therefore exclude that the genetic patchiness observed for these populations could reflect recruitment events from different source populations at different times, since the genetic patchiness in marine spawners is known to be ephemeral and temporally fluctuating [78]. Further genetic studies, based on collections across different years, are needed to definitely confirm or discard this last hypothesis, as obtained in previous genetic studies on other marine spawners (e.g. [79, 80]). Indeed, future studies aiming at disentangling the interplay among the possible drivers of CGP in *P. marmoratus* should rely on the resolving power offered by genomics, which allow researchers to depict the effects of biotic and abiotic factors on population dynamics through a seascape genomic approach.

## Methods

### Study species

*Pachygrapsus marmoratus* (Fabricius, 1787) is an intertidal crab belonging to the family Grapsidae (Crustacea; Brachyura). It is a dominant species in the supratidal fringe and at the eulittoral of rocky shores [52]. It is also abundant in harbours and marinas, showing a high resilience to anthropogenic disturbance [47]. *P. marmoratus* adults are relatively sedentary and are faithful to a small activity area [52]. Thus, dispersion and population connectivity are exclusively guaranteed by the larval planktonic stages, which persist in the open waters for about 4 weeks [53]. This species breeds from late April to late September, depending on the geographical area [81–84]. Spawning and settlement presumably peak around the new and full moon (spring tides) [83]. This rhythmicity, common to many species of brachyuran crabs, is generally under the control of a biological clock [85–87].

### Study area, sample collection and DNA extraction

We collected a total of 321 males and females *P. marmoratus* from four sites in the Ligurian Sea: Porto Mediceo of Livorno (PM, *N* = 80), Secche della Meloria (SM, *N* = 81), Le Grazie (LG, *N* = 80) and Riomaggiore (RM, *N* = 80) (Fig. 1, Table 1). PM is within the Livorno harbour, while SM is a small islet about 3 miles offshore of Livorno, established as a Marine Protected Area (MPA) in 2009. RM is a small pebble and rocky shore within the Cinque Terre National Park, and LG is a small marina, close to La Spezia harbour (Fig. 1).

A pereiopod was detached from each crab and preserved in absolute ethanol. DNA was extracted by overnight digestion at 55 °C in a lysis buffer and proteinase K, followed by isopropanol-ethanol precipitation [88]. Samples were resuspended in DNAase-free water and preserved at − 20 °C.

### Genetic analyses

Allelic variation at 11 microsatellite loci was determined using primers described for *P. marmoratus* by Fratini et al. [45, 47, 89]. Each locus was PCR-amplified in 10 μl total reaction using 1X reaction buffer, 1.5 mM of MgCl_2_, 0.5 μM of each primer, 200 μM of each dNTP and 0.5 U of Taq polymerase (Invitrogen). Thermal profiles consisted of an initial denaturation step of 5 min at 94 °C, 35 cycles of 60 s at 94 °C, annealing for 45 s at 54–57 °C (i.e. specific annealing temperature for each locus are reported in [45, 47, 89]) and extension for 60 s at 72 °C, and a final extension for 7 min at 72 °C.

PCR products were then pooled into four multilocus pools: pool P1 including loci Pm-79, Pm-109 and Pm-101; pool P2 including Pm-108, Pm-183 and Pm-99; pool P3 including Pm-16, Pm-86 and Pm-187; and pool P4 including Pm-84 and Pm-6. Amplicons were resolved by capillary electrophoresis in an Applied Biosystems 3130xl Genetic Analyzer and allele sizes scored against a GeneScan500 LIZ size standard using GeneMapper 5.0 (Applied Biosystems).

### Statistical analysis

#### Genetic diversity

Microsatellite alleles were checked for scoring errors due to stuttering, allele dropout and evidence of null alleles using Microchecker 2.2.3 [90]. Since individuals homozygous for a null allele or heterozygous for two null alleles appear as missing data, there may be an association between the amount of missing data at a locus in a population and deviation from HWE when null alleles are present [25]. Since all populations showed heterozygote deficiencies, we performed a linear regression between the absolute value of the difference between expected and observed heterozygosity and the proportion of individuals which failed to amplify at each locus, in order to understand if homozygote excess could be due to inbreeding or null alleles.

Since the outcome of our analysis indicated the occurrence of null alleles, we used the software FreeNA (www.montpellier.inra.fr/URLB) to calculate allele frequencies corrected for null alleles following the INA method described in Chapuis and Estoup [57]. The new corrected dataset was then used for all further analyses of population genetic variability and structure patterns.

The number of alleles and allelic richness for each locus and population were calculated using FSTAT 2.9.3.2 [91]. Linkage disequilibrium among loci was assessed for each population, each locus and each allele pairs across loci using FSTAT 2.9.3.2 [91]. For each pair of loci analysed, the software computes a correlation coefficient *r*^2^ over the entire population and for each population.

Observed and expected heterozygosity values as well as deviations from expectations of HWE were assessed for each population and each locus using ARLEQUIN 3.5 [92]. The test of HWE was an exact test employing a Markov chain. The number of steps after burn-in was set to 1,000,000 with 100,000 dememorization steps [92]. Significance levels were adjusted for multiple tests by using a sequential Bonferroni correction [93].

#### Population assignment and pairwise relatedness

To investigate whether there was a higher relatedness within or across the sampled population, we compared the mean pairwise relatedness calculated among individuals for each sampling site to average relatedness estimated across sampling sites using the Lynch and Ritland estimator [59] implemented in GenAlEx 6.5 [94]. Statistical significance was obtained after 1000 permutations of genotypes. GenAlEx was also used to assess population assignments following the frequency-based methods of Paetkau et al. [95].

#### Population structure

Genetic divergence among sampling sites was estimated using the exact test for population differentiation implemented in GENEPOP'007 [96]. This test verifies the existence of differences in allele frequencies at each locus and for each population. Single locus significance *P*-values were calculated using a Markov chain with 1,000 batches and 1,000 iterations per batch combined over loci using the Fisher method [97]. Genetic differentiation was also assessed by the *F*_ST_ estimator *θ* using ARLEQUIN 3.5 [92]. Statistical significance of *θ* values under the null hypothesis of no differentiation among sampling sites was assessed after 10,000 allele permutations.

To infer the number of genetic clusters (K) present in the microsatellite dataset, two clustering methods were applied: a Bayesian clustering approach (STRUCTURE, [98]) and a multivariate discriminant analysis of principal components (DAPC, [99]). The two approaches rely on different assumptions: STRUCTURE seeks groups in HWE and assumes the absence of linkage disequilibrium among loci within populations. Conversely, DAPC is free from assumptions about HWE or linkage disequilibrium, and then it is a most powerful test in unraveling genetic structuring.

The Bayesian-model clustering method implemented in STRUCTURE 2.3.4 [98] was used to infer the most likely number of genetically distinct clusters (populations) given the observed genotypes and to evaluate the proportion of each individual’s genotype belonging to each inferred population. We used the admixture model, which is the most appropriate for populations undergoing high rates of gene flow that may allow individuals to have mixed ancestry (i.e. recent ancestors from more than one population) [98]. We run 1,000,000 Markov Chain Monte Carlo (MCMC) iterations without prior population information for a *K* number of populations, ranging from 1 to 6, using a burn-in period of 20,000 iterations. We calculated the mean likelihood over 20 runs for each value of *K* using the correlated allele frequencies model, which provides greater power than the independent allele frequencies model, to detect distinct populations that are particularly closely related [100], and estimated the most likely number of clusters as described in Evanno et al. [60]. The *K* value with the highest Δ*K*, calculated by STRUCTURE Harvester, was then used as prior information to estimate the proportion of membership of each genotype in each of the *K* populations. Replicate runs for the same *K* were clustered and averaged to evaluate major clustering patterns using the CLUMPAK server [101]. Results were graphically visualized using STRUCTURE PLOT [102].

The DAPC analysis was performed using ADEGENET version 1.2.8 [103] in the R statistical environment R 3.2.2. DAPC is a methodological approach that requires data transformation using a principal component analysis (PCA) as a prior step to a discriminant analysis (DA). The DA analysis minimizes the genetic variation within groups and maximizes variation among groups at a given value of genetic clusters (K) [99]. The optimal number of genetic clusters *K* describing the data was identified using Bayesian information criterion (BIC) scores and the “find.clusters” function. In this analysis, the optimal *K* is expected to be associated with a low BIC score positioned along the BIC curve where the following BIC scores either increase or are only slightly lower than the chosen BIC value. To not overfit the discriminant function, we chose the optimal number of principal components for the DAPC using the “optim.a.score” function. The a-score captures the trade-off between the power of discrimination and overfitting using too many principal components in the analysis by measuring the proportion of successful reassignments of the DAPC analysis compared to K-means clustering (observed discrimination) and random clustering (random discrimination) [104]. We ran DAPC using all the available discriminant functions and calculated the assignment probability of individuals to each cluster, which were graphically visualized with a “compoplot” (i.e., a bar plot in which each individual is assigned to a particular cluster) using ADEGENET [104].

#### Contemporary migration patterns

In order to detect source-sink dynamics among our Ligurian Sea populations, we estimated recent migration rates using BayesAss 1.3 [105]. Based on the direction of main currents within the study area a pattern of unidirectional northward gene flow should have been predicted. Otherwise, under a CGP scenario we presumed to record migration rates not related to geographical features. The software implements a Bayesian approach using MCMC techniques to estimate the proportion of migrants/non-migrants in a population over the last few generations. The method does not assume that populations are at genetic equilibrium. The run consisted of 3 × 10^6^ iterations with a sampling frequency of 2,000 and the first 1 × 10^6^ steps discarded as burn-in. We used default setting delta values for allele frequencies, migration rates and inbreeding coefficients (i.e dM = 0.15, dA = 0.15, dF = 0.15).

## Supplementary information

**Additional file 1.** Dataset supporting the conclusions of this article (text file formatted as Genepop input).

**Additional file 2: Figure S1.** Optimal number of clusters defined by Evanno et al. (2005) ∆K (A, K = 2) and the Bayesian Information Content (BIC) value (B, K = 4).

**Additional file 3: Table S1.** Linkage disequilibrium among loci assessed for the totality of populations using FSTAT 2.9.3.2 (Goudet 1995). For each pair of loci analysed, the software computes a correlation coefficient r^2^ over the entire population and for each population. A threshold r^2^ value above 0.8 indicates a non-random association between the alleles present at two genetic loci (Carlson et al. 2004). Significant *P*-values (after Bonferroni corrections at *P* < 0.001) are also reported.

## Data Availability

The datasets supporting the conclusions of this article are included within the article and in the Additional file 1.

## Abbreviations

BIC: Bayesian information criterion
CGP: Chaotic Genetic Patchiness
DAPC: Discriminant Analysis of Principal Components
DNA: Deoxyribonucleic acid
dNTPs: Deoxynucleotide triphosphates
HWE: Hardy-Weinberg equilibrium
LG: Le Grazie
MCMC: Markov Chain Monte Carlo
MPA: Marine Protected Area
PCR: Polymerase Chain Reaction
PM: Porto Mediceo of Livorno
RM: Riomaggiore
SM: Secche della Meloria

## References

[1] Botsford LW, Hastings A, Gaines SD (2001). Dependence of sustainability on the configuration of marine reserves and larval dispersal distance. Ecol Lett.

[2] Cowen RK, Paris CB, Srinivasan A (2006). Scaling of connectivity in marine populations. Science..

[3] Cowen RK, Sponaugle S (2009). Larval dispersal and marine population connectivity. Annu Rev Mar Sci.

[4] Gagnaire PA, Broquet T, Aurelle D, Viard F, Souissi A, Bonhomme F (2015). Using neutral, selected, and hitchhiker loci to assess connectivity of marine populations in the genomic era. Evol Appl.

[5] Selkoe KA, Aloia CC, Crandall ED, Iacchei M, Liggins L, Puritz JB (2016). A decade of seascape genetics: contributions to basic and applied marine connectivity. Mar Ecol Prog Ser.

[6] Levin LA (2006). Recent progress in understanding larval dispersal: new directions and digressions. Integr Comp Biol.

[7] Hellberg ME, Burton RS, Neigel JE, Palumbi SR (2002). Genetic assessment of connectivity among marine populations. Bull Mar Sci.

[8] Neigel JE (1997). A comparison of alternative strategies for estimating gene flow from genetic markers. Annu Rev Ecol Syst.

[9] Palumbi SR (2004). Marine reserves and ocean neighborhoods: the spatial scale of marine populations and their management. Annu Rev Environ Resour.

[10] White C, Selkoe KA, Watson J, Siegel DA, Zacherl DC, Toonen RJ (2010). Ocean currents help explain population genetic structure. Proc R Soc B.

[11] Gaines SD, Gaylord B, Largier JL (2003). Avoiding current oversights in marine reserve design. Ecol Appl.

[12] Johnson M, Black R (1982). Chaotic genetic patchiness in an intertidal limpet, *Siphonaria* sp. Mar Biol.

[13] Hellberg ME (2009). Gene flow and isolation among populations of marine animals. Annu Rev Ecol Evol Syst.

[14] Aglieri G, Papetti C, Zane L, Milisenda G, Boero F, Piraino S (2014). First evidence of inbreeding, relatedness and chaotic genetic patchiness in the holoplanktonic jellyfish *Pelagia noctiluca* (Scyphozoa, Cnidaria). PLoS One.

[15] Arnaud-Haond S, Vonau V, Rouxel C, Bonhomme F, Prou J, Goyard E (2008). Genetic structure at different spatial scales in the pearl oyster (*Pinctada margaritifera cumingii*) in French Polynesian lagoons: beware of sampling strategy and genetic patchiness. Mar Biol.

[16] Banks SC, Piggott MP, Williamson JE, Bové U, Holbrook NJ, Beheregaray LB (2007). Oceanic variability and coastal topography shape genetic structure in a long-dispersing sea urchin. Ecology..

[17] Cornwell BH, Fisher JL, Morgan SG, Neigel JE (2016). Chaotic genetic patchiness without sweepstakes reproduction in the shore crab *Hemigrapsus oregonensis*. Mar Ecol Prog Ser.

[18] Doherty PJ, Planes S, Mather P (1995). Gene flow and larval duration in seven species of fish from the great barrier reef. Ecology..

[19] Hedgecock D (1994). Temporal and spatial genetic structure of marine animal populations in the California current. Cal Coop Ocean Fish.

[20] Hedgecock D, Beaumont A (1994). Does variance in reproductive success limit effective population sizes of marine organisms. Genetics and evolution of aquatic organisms.

[21] Johnson M, Black R (2006). Islands increase genetic subdivision and disrupt patterns of connectivity of intertidal snails in a complex archipelago. Evolution..

[22] Larson RJ, Julian RM (1999). Spatial and temporal genetic patchiness in marine populations and their implications for fisheries management. Cal Coop Ocean Fish.

[23] Muths D, Jollivet D, Gentil F, Davoult D (2009). Large-scale genetic patchiness among NE Atlantic populations of the brittle star *Ophiothrix fragilis*. Aquat Biol.

[24] Selkoe KA, Gaines SD, Caselle JE, Warner RR (2006). Current shifts and kin aggregation explain genetic patchiness in fish recruits. Ecology..

[25] Selwyn JD, Hogan JD, Downey-Wall AM, Gurski LM, Portnoy DS, Heath DD (2016). Kin-aggregations explain chaotic genetic patchiness, a commonly observed genetic pattern, in a marine fish. PLoS One.

[26] Villacorta-Rath C, Souza CA, Murphy NP, Green BS, Gardner C, Strugnell JM (2018). Temporal genetic patterns of diversity and structure evidence chaotic genetic patchiness in a spiny lobster. Mol Ecol.

[27] Broquet T, Viard F, Yearsley JM (2013). Genetic drift and collective dispersal can result in chaotic genetic patchiness. Evolution..

[28] Eldon B, Riquet F, Yearsley J, Jollivet D, Broquet T (2016). Current hypotheses to explain genetic chaos under the sea. Curr Zool.

[29] Hedgecock D, Barber PH, Edmands S (2007). Genetic approaches to measuring connectivity. Oceanography..

[30] Johnson MS, Black R (1984). Pattern beneath the chaos: the effect of recruitment on genetic patchiness in an intertidal limpet. Evolution..

[31] Johannesson K, Tatarenkov A (1997). Allozyme variation in a snail (*Littorina saxatilis*) - deconfounding the effects of microhabitat and gene flow. Evolution..

[32] Schmidt PS, Rand DM (1999). Intertidal microhabitat and selection at MPI: interlocus contrasts in the northern acorn barnacle, *Semibalanus balanoides*. Evolution.

[33] Schmidt PS, Rand DM (2001). Adaptive maintenance of genetic polymorphism in an intertidal barnacle: habitat-and life-stage-specific survivorship of MPI genotypes. Evolution..

[34] Pechenik JA (1999). On the advantages and disadvantages of larval stages in benthic marine invertebrate life cycles. Mar Ecol Prog Ser.

[35] Kordos L, Burton R (1993). Genetic differentiation of Texas Gulf Coast populations of the blue crab *Callinectes sapidus*. Mar Biol.

[36] Hendry AP, Day T (2005). Population structure attributable to reproductive time: isolation by time and adaptation by time. Mol Ecol.

[37] Jolly MT, Thiébaut E, Guyard P, Gentil F, Jollivet D (2014). Meso-scale hydrodynamic and reproductive asynchrony affects the source–sink metapopulation structure of the coastal polychaete *Pectinaria koreni*. Mar Biol.

[38] Hedgecock D, Pudovkin AI (2011). Sweepstakes reproductive success in highly fecund marine fish and shellfish: a review and commentary. Bull Mar Sci.

[39] Christie MR, Johnson DW, Stallings CD, Hixon MA (2010). Self-recruitment and sweepstakes reproduction amid extensive gene flow in a coral-reef fish. Mol Ecol.

[40] Liu JX, Ely B (2009). Sibship reconstruction demonstrates the extremely low effective population size of striped bass *Morone saxatilis* in the Santee–Cooper system, South Carolina. USA Mol Ecol.

[41] Yearsley JM, Viard F, Broquet T (2013). The effect of collective dispersal on the genetic structure of a subdivided population. Evolution..

[42] Siegel D, Mitarai S, Costello C, Gaines S, Kendall B, Warner R (2008). The stochastic nature of larval connectivity among nearshore marine populations. Proc Natl Acad Sci.

[43] Deli T, Bahles H, Said K, Chatti N (2015). Patterns of genetic and morphometric diversity in the marbled crab (*Pachygrapsus marmoratus*, Brachyura, Grapsidae) populations across the Tunisian coast. Acta Oceanol Sin.

[44] Deli T, Fratini S, Ragionieri L, Said K, Chatti N, Schubart CD (2016). Phylogeography of the marbled crab *Pachygrapsus marmoratus* (Decapoda, Grapsidae) along part of the African Mediterranean coast reveals genetic homogeneity across the Siculo-Tunisian Strait versus heterogeneity across the Gibraltar Strait. Mar Biol Res.

[45] Fratini S, Ragionieri L, Cutuli G, Vannini M, Cannicci S (2013). Pattern of genetic isolation in the crab *Pachygrapsus marmoratus* within the Tuscan archipelago (Mediterranean Sea). Mar Ecol Prog Ser.

[46] Fratini S, Schubart CD, Ragionieri L, Held C, Koenemann S, Schubart CD (2011). Population genetics in the rocky shore crab *Pachygrapsus marmoratus* from the western Mediterranean and eastern Atlantic: complementary results from mtDNA and microsatellites at different geographic scales. Phylogeography and population genetics in Crustacea.

[47] Fratini S, Zane L, Ragionieri L, Vannini M, Cannicci S (2008). Relationship between heavy metal accumulation and genetic variability decrease in the intertidal crab *Pachygrapsus marmoratus* (Decapoda; Grapsidae). Estuar Coast Shelf S.

[48] Kalkan E, Karhan SÜ, Bilgin R (2013). Population genetic structure of the marbled crab, *Pachygrapsus marmoratus* from Turkish coasts of the Black Sea and the eastern Mediterranean. Rapports et procès verbaux des réunions, Commission internationale pour l'exploration scientifique de la Mer Méditerranée.

[49] Silva IC, Mesquita N, Schubart CD, Alves MJ, Paula J (2009). Genetic patchiness of the shore crab *Pachygrapsus marmoratus* along the Portuguese coast. J Exp Mar Biol Ecol.

[50] Fratini S, Ragionieri L, Deli T, Harrer A, Marino IA, Cannicci S (2016). Unravelling population genetic structure with mitochondrial DNA in a notional panmictic coastal crab species: sample size makes the difference. BMC Evol Biol.

[51] Pascual M, Rives B, Schunter C, Macpherson E (2017). Impact of life history traits on gene flow: a multispecies systematic review across oceanographic barriers in the Mediterranean Sea. PLoS One.

[52] Cannicci S, Paula J, Vannini M (1999). Activity pattern and spatial strategy in *Pachygrapsus marmoratus* (Decapoda: Grapsidae) from Mediterranean and Atlantic shores. Mar Biol.

[53] Cuesta JA, Rodríguez A (2000). Zoeal stages of the intertidal crab *Pachygrapsus marmoratus* (Fabricius, 1787) (Brachyura, Grapsidae) reared in the laboratory. Hydrobiologia..

[54] Cattaneo-Vietti R, Albertelli G, Aliani S, Bava S, Bavestrello G, Cecchi LB (2010). The Ligurian Sea: present status, problems and perspectives. Chem Ecol.

[55] Sciascia R, Magaldi MG, Vetrano A (2019). Current reversal and associated variability within the Corsica Channel: the 2004 case study. Deep-Sea Res Pt I.

[56] Prieur L, D'ortenzio F, Taillandier V, Testor P, Migon C, Nival P, Sciandra A (2020). Physical oceanography of the Ligurian Sea. The Mediterranean Sea in the era of global change 1: 30 years of multidisciplinary study of the Ligurian Sea. Wiley online library.

[57] Chapuis M-P, Estoup A (2006). Microsatellite null alleles and estimation of population differentiation. Mol Biol Evol.

[58] Carlson CS, Eberle MA, Rieder MJ, Yi Q, Kruglyak L, Nickerson DA (2004). Selecting a maximally informative set of single-nucleotide polymorphisms for association analyses using linkage disequilibrium. Am J Hum Genet.

[59] Lynch M, Ritland K (1999). Estimation of pairwise relatedness with molecular markers. Genetics..

[60] Evanno G, Regnaut S, Goudet J (2005). Detecting the number of clusters of individuals using the software STRUCTURE: a simulation study. Mol Ecol.

[61] Marko PB, Hart MW, Tyler C, Adam R, Andreas H (2018). Genetic analysis of larval dispersal, gene flow, and connectivity. Evolutionary ecology of marine invertebrate larvae.

[62] Smith P, McVeagh S, Won Y, Vrijenhoek R (2004). Genetic heterogeneity among New Zealand species of hydrothermal vent mussels (Mytilidae: *Bathymodiolus*). Mar Biol.

[63] Watts R, Johnson M, Black R (1990). Effects of recruitment on genetic patchiness in the urchin *Echinometra mathaei* in Western Australia. Mar Biol.

[64] Zouros E (1984). Possible explanations of heterozygosity deficiency in bivalve molluscs. Malacologia..

[65] Carson HS, López-Duarte PC, Rasmussen L, Wang D, Levin LA (2010). Reproductive timing alters population connectivity in marine metapopulations. Curr Biol.

[66] Treml EA, Ford JR, Black KP, Swearer SE (2015). Identifying the key biophysical drivers, connectivity outcomes, and metapopulation consequences of larval dispersal in the sea. Mov Ecol.

[67] Treml EA, Roberts JJ, Chao Y, Halpin PN, Possingham HP, Riginos C (2012). Reproductive output and duration of the pelagic larval stage determine seascape-wide connectivity of marine populations. Integr Comp Biol.

[68] Fratini S, Ragionieri L, Cannicci S (2016). Demographic history and reproductive output correlates with intraspecific genetic variation in seven species of indo-Pacific mangrove crabs. PLoS One.

[69] Morgan SG, Fisher JL (2010). Larval behavior regulates nearshore retention and offshore migration in an upwelling shadow and along the open coast. Mar Ecol Prog Ser.

[70] Morgan SG, Fisher JL, Miller SH, McAfee ST, Largier JL (2009). Nearshore larval retention in a region of strong upwelling and recruitment limitation. Ecology..

[71] Pedrotti ML, Fenaux L (1992). Dispersal of echinoderm larvae in a geographical area marked by upwelling (Ligurian Sea, NW Mediterranean). Mar Ecol Prog Ser.

[72] Ottmann D, Grorud-Colvert K, Sard NM, Huntington BE, Banks MA, Sponaugle S (2016). Long-term aggregation of larval fish siblings during dispersal along an open coast. Proc Natl Acad Sci.

[73] St-Onge P, Tremblay R, Sévigny J-M (2015). Tracking larvae with molecular markers reveals high relatedness and early seasonal recruitment success in a partially spawning marine bivalve. Oecologia..

[74] Veliz D, Duchesne P, Bourget E, Bernatchez L (2006). Genetic evidence for kin aggregation in the intertidal acorn barnacle (*Semibalanus balanoides*). Mol Ecol.

[75] Costantini F, Fauvelot C, Abbiati M (2007). Genetic structuring of the temperate gorgonian coral (*Corallium rubrum*) across the western Mediterranean Sea revealed by microsatellites and nuclear sequences. Mol Ecol.

[76] McFadden CS, Aydin K (1996). Spatial autocorrelation analysis of small-scale genetic structure in a clonal soft coral with limited larval dispersal. Mar Biol.

[77] Raffaelli D, Hawkins SJ (2012). Intertidal ecology.

[78] Ward RD (2006). The importance of identifying spatial population structure in restocking and stock enhancement programmes. Fish Res.

[79] Toonen RJ, Grosberg RK, Held C, Koenemann S, Schubart CD (2011). Causes of chaos: spatial and temporal genetic heterogeneity in the intertidal anomuran crab *Petrolisthes cinctipes*. Phylogeography and population genetics in Crustacea.

[80] Calderón I, Palacín C, Turon X (2009). Microsatellite markers reveal shallow genetic differentiation between cohorts of the common sea urchin *Paracentrotus lividus* (Lamarck) in Northwest Mediterranean. Mol Ecol.

[81] Flores AA, Paula J (2002). Population dynamics of the shore crab *Pachygrapsus marmoratus* (Brachyura: Grapsidae) in the central Portuguese coast. J Mar Biol Assoc U K.

[82] Ingle RW (1980). British crabs.

[83] Vernet-Cornubert G (1958). Recherches Sur la sexualité du crabe *Pachygrapsus marmoratus* (Fabricius). Arch Zool Exp Gen.

[84] Zariquiey AR (1968). Crustáceos decápodos ibéricos. Investig Pesq.

[85] Christy JH (2011). Timing of hatching and release of larvae by brachyuran crabs: patterns, adaptive significance and control. Integr Comp Biol.

[86] Morgan SG, Christy JH (1995). Adaptive significance of the timing of larval release by crabs. Am Nat.

[87] Skov MW, Hartnoll RG, Ruwa RK, Shunula JP, Vannini M, Cannicci S (2005). Marching to a different drummer: crabs synchronize reproduction to a 14-month lunar-tidal cycle. Ecology..

[88] Sambrook J, Russell DW (2001). Molecular cloning: a laboratory manual.

[89] Fratini S, Ragionieri L, Papetti C, Pitruzzella G, Rorandelli R, Barbaresi S (2006). Isolation and characterization of microsatellites in *Pachygrapsus marmoratus* (Grapsidae; Decapoda; Brachyura). Mol Ecol Notes.

[90] Van Oosterhout C, Hutchinson WF, Wills DP, Shipley P (2004). MICRO-CHECKER: software for identifying and correcting genotyping errors in microsatellite data. Mol Ecol Notes.

[91] Goudet J (1995). FSTAT (version 1.2): a computer program to calculate F-statistics. J Hered.

[92] Excoffier L, Lischer HE (2010). Arlequin suite ver 3.5: a new series of programs to perform population genetics analyses under Linux and windows. Mol Ecol Resour.

[93] Rice WR (1989). Analyzing tables of statistical tests. Evolution..

[94] Peakall R, Smouse PE (2006). GENALEX 6: genetic analysis in excel. Population genetic software for teaching and research. Mol Ecol Notes.

[95] Paetkau D, Slade R, Burden M, Estoup A (2004). Genetic assignment methods for the direct, real-time estimation of migration rate: a simulation-based exploration of accuracy and power. Mol Ecol.

[96] Rousset F (2008). Genepop’007: a complete re-implementation of the genepop software for windows and Linux. Mol Ecol Resour.

[97] Raymond M, Rousset F (1995). An exact test for population differentiation. Evolution..

[98] Pritchard JK, Stephens M, Donnelly P (2000). Inference of population structure using multilocus genotype data. Genetics..

[99] Jombart T, Devillard S, Balloux F (2010). Discriminant analysis of principal components: a new method for the analysis of genetically structured populations. BMC Genet.

[100] Porras-Hurtado L, Ruiz Y, Santos C, Phillips C, Carracedo Á, Lareu M (2013). An overview of STRUCTURE: applications, parameter settings, and supporting software. Front Genet.

[101] Kopelman NM, Mayzel J, Jakobsson M, Rosenberg NA, Mayrose I (2015). Clumpak: a program for identifying clustering modes and packaging population structure inferences across K. Mol Ecol Resour.

[102] Ramasamy RK, Ramasamy S, Bindroo BB, Naik VG (2014). STRUCTURE PLOT: a program for drawing elegant STRUCTURE bar plots in user friendly interface. SpringerPlus..

[103] Jombart T (2008). *adegenet*: a R package for the multivariate analysis of genetic markers. Bioinformatics..

[104] Jombart T, Collins C (2015). A tutorial for discriminant analysis of principal components (DAPC) using adegenet 2.0. 0.

[105] Wilson GA, Rannala B (2003). Bayesian inference of recent migration rates using multilocus genotypes. Genetics..

