# Supplementary figures and images for: Investigation of mechanisms underlying chaotic genetic patchiness in the intertidal marbled crab Pachygrapsus marmoratus (Brachyura: Grapsidae) across the Ligurian Sea
Source: BMC Evol Biol. 2020 Aug 24;20:108. doi: 10.1186/s12862-020-01672-x (PMC7444255; doi:10.1186/s12862-020-01672-x)

**A**

$\Delta K = \frac{\text{mean}(|L''(K)|)}{\text{sd}(L(K))}$

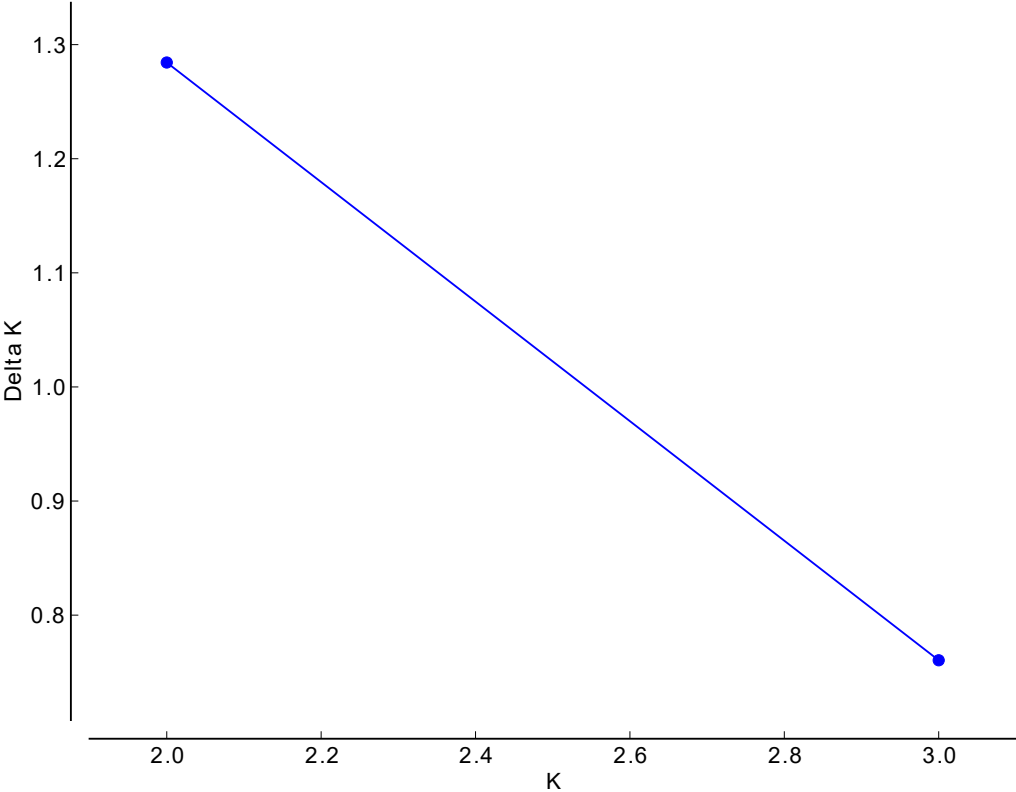

**B**

**Value of BIC  
versus number of clusters**

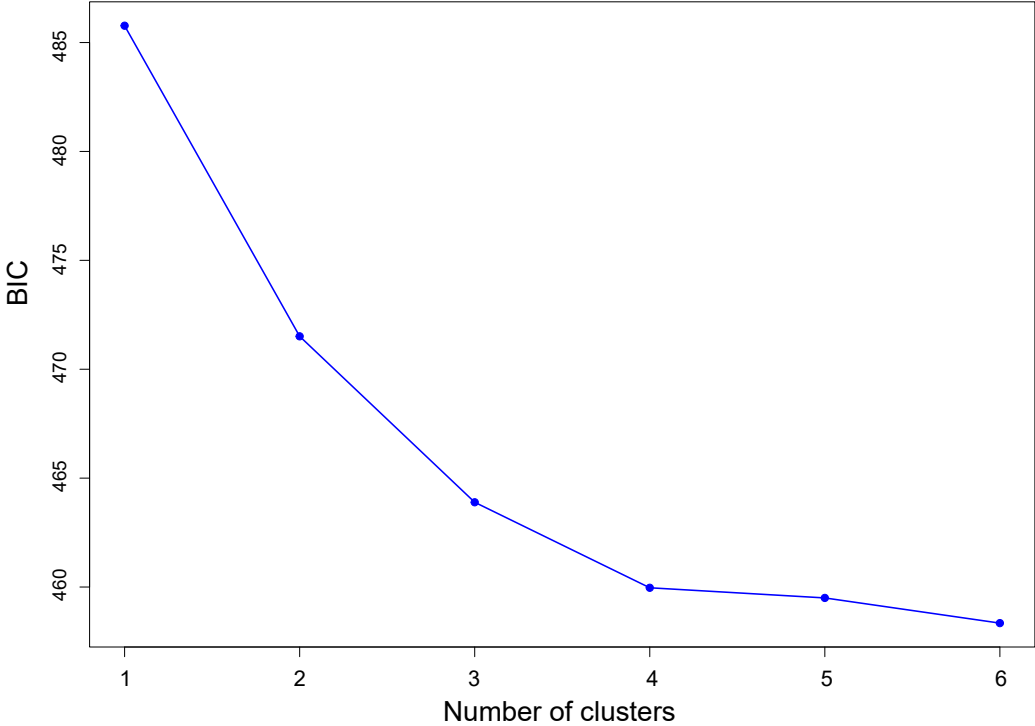

Supplement: Supplementary file 2 — Additional file 2: Figure S1. Optimal number of clusters defined by Evanno et al. (2005) ∆K (A, K = 2) and the Bayesian Information Content (BIC) value (B, K = 4). [file 12862_2020_1672_MOESM2_ESM.pdf]
