# Supplementary material for: Investigation of mechanisms underlying chaotic genetic patchiness in the intertidal marbled crab Pachygrapsus marmoratus (Brachyura: Grapsidae) across the Ligurian Sea
Source: BMC Evol Biol. 2020 Aug 24;20:108. doi: 10.1186/s12862-020-01672-x (PMC7444255; doi:10.1186/s12862-020-01672-x)
Supplement: Supplementary file 3 — Additional file 3: Table S1. Linkage disequilibrium among loci assessed for the totality of populations using FSTAT 2.9.3.2 (Goudet 1995). For each pair of loci analysed, the software computes a correlation coefficient r2 over the entire population and for each population. A threshold r2 value above 0.8 indicates a non-random association between the alleles present at two genetic loci (Carlson et al. 2004). Significant P-values (after Bonferroni corrections at P < 0.001) are also reported. [file 12862_2020_1672_MOESM3_ESM.docx]

**Table S1** Linkage disequilibrium among loci assessed for the totality of populations using FSTAT 2.9.3.2 (Goudet 1995). For each pair of loci analysed, the software computes a correlation coefficient *r*^2^ over the entire population and for each population. A threshold *r*^2^ value above 0.8 indicates a non-random association between the alleles present at two genetic loci (Carlson et al. 2004). Significant P-values (after Bonferroni corrections at P < 0.001) are also reported.

| **Loci pair** | **Correlation coefficient (***r*^2^**)** | **P-value** |
| --- | --- | --- |
| 79 × 109-Bi | 0.01435 | 0.002 |
| 79 × 101 | 0.01489 | 0.878 |
| 79 × 108 | 0.00633 | 0.164 |
| 79 × 183 | 0.01178 | 0.802 |
| 79 × 99 | 0.01207 | 0.775 |
| 79 × 16 | 0.01583 | 0.512 |
| 79 × 187 | 0.01351 | 0.967 |
| 79 × 84 | 0.02375 | 0.171 |
| 79 × 6 | 0.00781 | 0.954 |
| 109-Bi × 101 | 0.01578 | 0.004 |
| 109-Bi × 108 | 0.01416 | 0.235 |
| 109-Bi × 183 | 0.01414 | 0.623 |
| 109-Bi × 99 | 0.01652 | 0.064 |
| 109-Bi × 16 | 0.01536 | 0.217 |
| 109-Bi × 187 | 0.01535 | 0.022 |
| 109-Bi × 84 | 0.01409 | 0.086 |
| 109-Bi × 6 | 0.0138 | 0.498 |
| 101 × 108 | 0.01345 | 0.906 |
| 101 × 183 | 0.01898 | 0.18 |
| 101 × 99 | 0.01593 | 0.676 |
| 101 × 16 | 0.01584 | 0.534 |
| 101 × 187 | 0.01517 | 0.05 |
| 101 × 84 | 0.01988 | 0.173 |
| 101 × 6 | 0.01707 | 0.046 |
| 108 × 183 | 0.01516 | 0.003 |
| 108 × 99 | 0.01737 | 0.592 |
| 108 × 16 | 0.01447 | 0.07 |
| 108 × 187 | 0.02 | 0.085 |
| 108 × 84 | 0.00679 | 0.144 |
| 108 × 6 | 0.01399 | 0.115 |
| 183 × 99 | 0.01281 | 0.715 |
| 183 × 16 | 0.01131 | 0.503 |
| 183 × 187 | 0.01243 | 0.179 |
| 183 × 84 | 0.01915 | 0.191 |
| 183 × 6 | 0.17557 | 0.001 |
| 99 × 16 | 0.01568 | 0.613 |
| 99 × 187 | 0.01626 | 0.843 |
| 99 × 84 | 0.01558 | 0.01 |
| 99 × 6 | 0.01263 | 0.788 |
| 16 × 187 | 0.01768 | 0.071 |
| 16 × 84 | 0.00934 | 0.185 |
| 16 × 6 | 0.01415 | 0.654 |
| 187 × 84 | 0.01619 | 0.319 |
| 187 × 6 | 0.00853 | 0.57 |
| 84 × 6 | 0.01273 | 0.65 |

**References**

Carlson CS, Eberle MA, Rieder MJ, Yi Q, Kruglyak L, Nickerson DA. Selecting a maximally informative set of single-nucleotide polymorphisms for association analyses using linkage disequilibrium. Am J Hum Genet. 2004;74:106-20.

Goudet J. FSTAT (version 1.2): a computer program to calculate F-statistics. J Hered. 1995;86:485-6.
